# Multiplex PCR of bronchoalveolar lavage fluid in children enhances the rate of pathogen detection

**DOI:** 10.1186/s12890-019-0894-7

**Published:** 2019-07-18

**Authors:** Eva Tschiedel, Arkadius Goralski, Jörg Steinmann, Peter-Michael Rath, Margarete Olivier, Uwe Mellies, Tanja Kottmann, Florian Stehling

**Affiliations:** 1 Department of Pediatrics I, University Duisburg-Essen, University Hospital Essen, Hufelandstr. 55, 45122 Essen, Germany; 2 Department of Pediatrics III, University Duisburg-Essen, University Hospital Essen, Hufelandstr. 55, 45122 Essen, Germany; 3 Institute for Medical Microbiology, University of Duisburg-Essen, Essen, Germany; 4 Institute of Clinical Hygiene, Medical Microbiology and Infectiology, Klinikum Nürnberg, Paracelsus Medical University, Nuremberg, Germany; 5 Private Institute of Medical Statistics, 59077 Hamm, Westfalen Germany

**Keywords:** Bronchoalveolar lavage, Multiplex PCR, Culture, Pneumonia, Children, Bronchoscopy

## Abstract

**Background:**

Culturing of bronchoalveolar lavage (BAL) fluid is a commonly used method for pathogen detection in pneumonia. However, the sensitivity is low, especially in patients pre-treated with anti-infective agents. The early detection of a pathogen is crucial for the outcome of respiratory tract infections. For bloodstream infections, a multiplex polymerase chain reaction (PCR) assay (SeptiFast®, SF) is available for improved pathogen detection from blood.

**Objective:**

The aim of the present study was to determine whether the SF assay is applicable to the BAL of children with pulmonary infections and whether the frequency of pathogen detection is enhanced by the use of this multiplex PCR method.

**Methods:**

We investigated 70 BAL samples of 70 children simultaneously by culture and multiplex PCR. The frequency of pathogen detection was compared.

**Results:**

Pathogens were detected more frequently by SF than by culture (83% vs. 31%; *p* < 0.001). This advantage was shown for immunocompetent patients (*p* = 0.001) as well as for immunocompromised patients (*p* = 0.003). The majority (38/44; 86%) of the Gram positive cocci were only detected by SF. Fungal organisms were detected in 7/70 patients (10%) by SF and in 2/70 (3%) by culture (*p* = 0.125).

**Conclusion:**

Compared to conventional culture, the use of the SF assay on the BAL of children with pneumonia increases pathogen detection rates and therefore adds important information to guide anti-infective therapy.

## Background

Severe bacterial and fungal infections in critically ill patients require immediate anti-infective treatment to reduce mortality [1, 2]. Bronchoalveolar lavage (BAL) is frequently performed in patients with severe pneumonia for microbiological workup. Usually, antibiotic treatment is started empirically, as the underlying infectious pathogen is unknown. Nevertheless it is crucial to modify/deescalate antibiotic treatment according to microbiological results to optimize efficacy and to reduce the side effects. For pneumonia and bloodstream infections, cultivation of underlying pathogens is the most commonly used method for pathogen detection. In children with pneumonia, the only method to obtain specimens from the lower airways is BAL [3]. However, BAL is a semi- invasive diagnostic procedure with a significant risk of deterioration in respiratory compromised children. Limitations of the microbiological workup of BAL samples include the low sensitivity of Gram staining [4] and cultures, especially after initiation of antibiotic treatment [5, 6], as well as the time until results are available (Gram staining 12 h; culture 24–48 h). Therefore, children with suspected pneumonia are commonly treated empirically with antibiotics. Only in the case of refractory infection or exceptional severity of an infection is BAL performed. Furthermore, BAL is recommended in immunocompromised children with pneumonia, especially if pulmonary aspergillosis is suspected [7]. As bronchoscopy and BAL in children with pneumonia are associated with an elevated risk for complications [8], examination of BAL fluid samples requires special attention.

In addition to culture another diagnostic tool based on the detection of infectious agents by molecular genetic methods is commercially available: a multiplex PCR-system (LightCycler® SeptiFast; SF) that detects 20 different types of bacteria and fungi. The results of multiplex PCR- systems are available within 6 h. For sepsis, it has been shown that multiplex PCR from blood increases the rate of pathogen detection from 17 to 20 to 24%–33% and shortens the time interval to diagnosis [9, 10]. Furthermore, multiplex PCR is more sensitive than culture in patients under antimicrobial treatment (3–10% up to 15–36%) [11, 12]. In adults, a pilot study suggests the same advantages for BAL fluid in patients with pneumonia [13]. In addition to galactomannan detection [14], PCR -based diagnostic systems are increasingly being used for the detection of *Aspergillus* from BAL because cultivation is challenging [15, 16]. *Aspergillus* detection has increased from 23 to 39% [17]. Furthermore, particular multiplex PCRs allow the detection of DNA fragments associated with azole resistance [18, 19].

The aim of the present study was to determine whether SF, which was designed and extensively validated for blood samples, is also applicable to BAL from children with pneumonia and whether the frequency of pathogen detection can be enhanced.

## Methods

During the period between September 2011 and May 2017, 70 samples of BAL fluid were taken from 70 non-ventilated children and young adults. For all patients pulmonary infection was suspected, and the indication for BAL was established. “Suspected pulmonary infection” was defined as persistent or threatening respiratory symptoms such as coughing or shortness of breath without another cause and/or matching radiological findings. BAL samples were simultaneously investigated by culture and SF. Patient age ranged between 0 and 25 years (median 6 years); 34 were male, and 36 were female.

Forty-one samples were drawn from patients who were defined as immunosuppressed (59%), 36 from patients (51%) under anti-infective treatment at the time of sampling.

The majority (89%) of patients suffered from an underlying disease, such as malignant illnesses with or without neutropenia, organ transplantation (liver, kidney, or bone marrow), chronic lung disease, and neuromuscular disease. Eight samples (11%) were taken from patients without underlying disease. The detailed characteristics of all patients are shown in Table 1.

**Table 1:** Patient characteristics

Underlying condition	No. of patients	Gender [f/m]	Age [y] median/range	Immunosuppressive patients [%]	Patients with anti-infective pre-treatment [%]
Patients with underlying haematological or oncological disease	29/70 (41%)	12/17	8/0–25	26/29 (90%)	20/30 (67%)
After bone marrow transplantation	6/70 (9%)	3/3	9/3–14	6/6 (100%)	5/6 (83%)
After solid organ transplantation	6/70 (9%)	5/1	6.5/1–14	6/6 (100%)	3/6 (50%)
Chronic lung disease	10/70 (14%)	7/3	9/0–14	1/10 (10%)	6/10 (60%)
Neuromuscular disease	3/70 (4%)	1/2	4/0–22	1/3 (33%)	0/3 (0%)
Other	5/70 (7%)	5/0	5/1–16	1/5 (20%)	1/5 (20%)
Previously healthy	11/70 (16%)	3/8	2.5/0–14	0/11 (0%)	1/11 (9%)
Total	70	36/34	6.5 (0–25)	41/70 (59%)	36/70 (51%)

*f* Female, *m* Male, *y* Years

Patients were defined as immunosuppressed when they were receiving chemotherapy for malignoma, when they were receiving medical immunosuppressive therapy after organ transplantation, or when they suffered from inborn immunodeficiency.

Pre-existing anti-infective treatment was defined as any antibiotic therapy during 24 h prior to BAL sampling.

Contamination of the sample was assumed when bacteria that do not usually cause pneumonia were detected in either the culture or SF. Contaminating organisms were *coagulase-negative staphylococci (conS)*, *E. faecalis* and *E. faecium, Streptococcus spp.* except for *S. pneumoniae,* and *Candida spp.*

Bronchoscopy was performed according to the ATS Standards [20]. After deep sedation was established, the airway was accessed via a nostril. The upper airways were explored, and the larynx was anaesthetized with 0.5 ml of 1% lidocaine using the spray as you go procedure [21]. Thereafter, the entire bronchial tree was visually examined. BAL was performed in the lobe where the major pathology was displayed radiographically or in the right middle lobe.

For BAL, the flexible bronchoscope was gently wedged into the selected bronchus, and up to four aliquots of 1 ml/kg normal saline were instilled via the suction channel. After installation, each aliquot was manually aspirated. The retrieved BAL fluid was immediately transferred to the microbiology laboratory for direct microscopy, culture and PCR. Microbiological testing was carried out according to the Quality Standards for the Microbiological Diagnosis of Infectious Diseases (MiQ). For cultures, we used common culture media (Columbia blood agar, Columbia agar with sheep blood, cooked blood agar, MacConkey-agar, Brilliance selective agar, malt extract agar, Oxoid Deutschland GmbH, Wesel, Germany), which were incubated at 37 °C for up to 72 h. Malt extract agar plates were further incubated at room temperature for up to 7 days. For pathogen identification, mass -spectrometry by VITEK® MS mass spectrometer was used and for susceptibility testing, a VITEK® 2 with VITEK® 2 AST cards (bioMerieux, Marcy l‘Etoile, France) was used.

For DNA detection, a commercially available test system (LightCycler® SeptiFast, Roche, Mannheim, Germany) was used according to the instructions of the manufacturer. For DNA-extraction, the MagNa pure compact nucleic acid isolation kit I was used in combination with the MagNa Pure compact platform [22] . SF is a molecular genetic test system capable of amplifying and detecting genomes of 20 different bacteria and fungi from blood by real-time multiplex PCR. This system can detect *E. coli, K. pneumoniae/oxytoca, S. marcescens, E. cloacae/aerogenes, P. mirabilis, P. aeruginosa, A. baumannii, S. maltophilia, S. aureus, conS, S. pneumoniae, Streptococcus spp., E. faecium, E. faecalis, C. albicans, C. tropicalis, C. parapsilosis, C. krusei, C. glabrata and A. fumigatus*.

### Clinical data were retrieved by retrospective chart review

Patient data were given as medians and ranges. Proportions of dichotomous related samples were tested with McNemar’s test. A value of *p* < 0.05 was considered significant.

The study protocol for the retrospective analysis was approved by the local ethics committee (15–6499-BO).

## Results

Seventy samples of 70 patients were examined. In total, in 59/70 patients (84% of patients), at least one potential pathogen was found. Pathogen detection was more frequent by SF (83%) than by culture (31%, *p* < 0.001; Table 2).

**Table 2:** Overall frequency of pathogen detection

	Culture -	Culture +	Total
SF -	11	1	12 (17%)
SF +	37	21	58 (82%)
Total	48 (69%)	22 (31%)	70

### Contamination

In 28 cases (40%) at least one of the pathogens detected by SF was *conS.*, *E. faecalis*/*faecium, Streptococcus spp.,* or *Candida spp.* These organisms were considered contaminants or clinically irrelevant. In 5 cases (7%), concordant with the SF results, these pathogens were found by culture. In 16 cases (23%) the assumed contaminating microbes were found in addition to potential pathogens in the SF test or culture. In this scenario, the sample was assessed as positive. After exclusion of all contaminants, pathogens were detected in 53/70 patients (76%). Pathogens were detected by SF in 67% of samples and by culture in 26% (*p* < 0.001) (Table 3). Considering all patients suffering from pneumonia, the sensitivity was 0.67 for SF, 0.26 for culture and 0.76 for the combination of both.

**Table 3:** Frequency of pathogen detection after exclusion of contaminations

	Culture -	Culture +	Total
SF-	17	6	23
SF+	35	12	47
Total	52	18	70

### Spectrum of detected pathogens

In total, 68 pathogens belonging to 11 different species were detected. Forty-four of the pathogens were Gram positive, 16 were Gram negative, and 8 were moulds (Table 4). Of the pathogens exclusively found by SF 38 were Gram positive (81%), 3 were Gram negative (6%), and 6 were moulds (13%). Of the pathogens that were only found by culture, 18% were Gram positive, 73% were Gram negative, and 9% were moulds. Correspondingly, identified pathogens were 40% Gram positive, 50% Gram negative and 10% moulds. Eighty-six percent of the Gram positive bacteria were found only by SF.

**Table 4:** Spectrum of detected pathogens

Pathogen	Only SF +	Only culture +	Both consistently +	Total
*S. aureus*	11	2	3	16
*S. pneumoniae*	27	0	1	28
*A. xylosoxidans* ^a^	0	1	0	1
*E. coli*	0	2	1	3
*Enterobacter spp.*	2	0	1	3
*H. influenzae* ^a^	0	4	0	4
*Klebsiella spp.*	0	0	2	2
*M. catarrhalis* ^a^	0	1	0	1
*S. maltophilia*	1	0	1	2
*A. fumigatus*	6	0	1	7
*P. variotii* ^a^	0	1	0	1
Total	47	11	10	68

^a^ *= not part of SF-spectrum*

#### Detection of bacteria

The SF test was positive for bacteria in 43/70 (61%) of patients whereas 17/70 (24%) of the cultures were positive (*p* < 0.001) for bacteria.

#### Detection of fungi

The SF test was positive for fungi in 7/70 (10%) of patients, whereas 2/70 (3%) of cultures were positive for fungi (*p* = 0.125). According to the definitions of the European Organization for Research and Treatment of Cancer/Invasive Fungal Infections Cooperative Group and the National Institute of Allergy and Infectious Diseases Mycoses Study Group (EORTC/MSG) Consensus Group [23], of the six patients with *Aspergillus* detected in the SF test, Aspergillosis was probable in 3 and possible in two patients. The only patient with concordant *Aspergillus* detection in the SF test and by culture suffered from CF. The patient with *P. variotii* found in culture had a possible invasive fungal infection.

### Pathogen detection in patients under immunosuppression vs. patients without immunosuppression

Forty-one samples (59%) were taken from patients under immunosuppression. In this subgroup the SF test was positive in 54% (22/41), and the culture was positive in 17% (7/41) (*p* = 0.003). In the immunocompetent patients (*n* = 29), the SF test was positive in 86% (25/29) and the culture positive in 38% (11/29) of the samples (*p* = 0.001).

#### Spectrum of detected pathogens in patients under immunosuppression

**Fig. 1:**
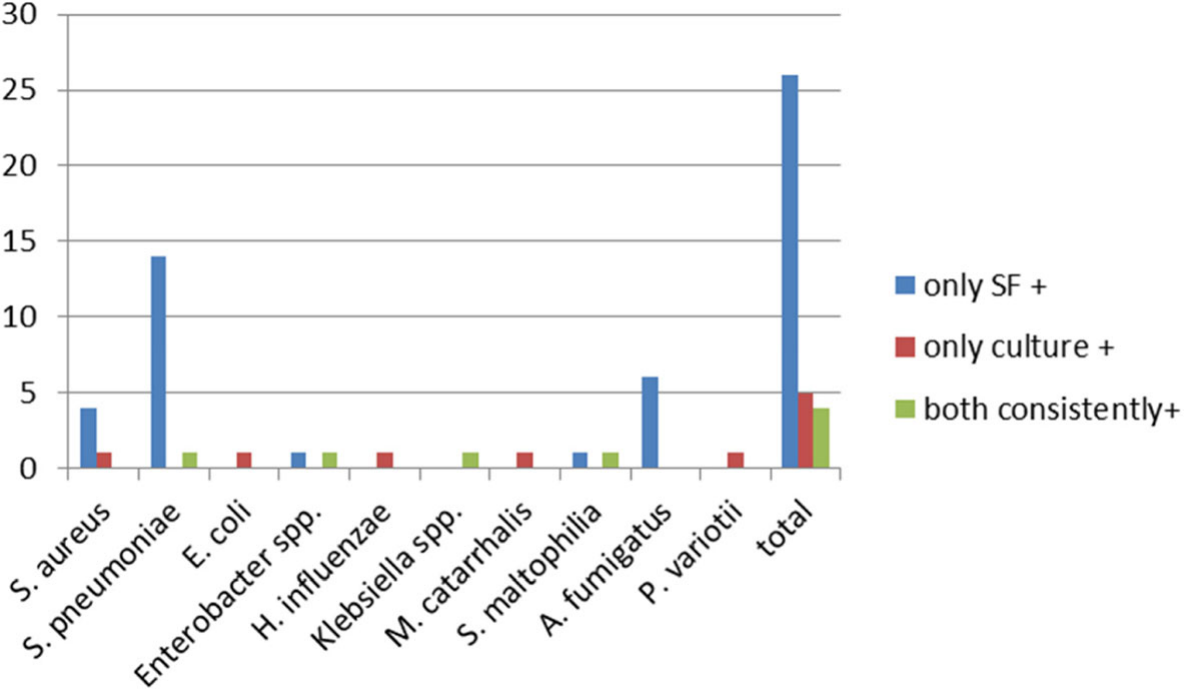
Spectrum of detected pathogens in patients under immunosuppression.  only SF +,  only culture +,  both consistently +

Here, 35 pathogens belonging to 10 different species were detected. Twenty of the pathogens were Gram positive, 8 Gram negative and 7 were moulds (Fig. 1). Of the pathogens exclusively found by SF, 18 were Gram positive (69%), 2 were Gram negative (8%), and 6 were moulds (23%). The pathogens that were exclusively positive in the cultures were 20% Gram positive, 60% Gram negative and 20% moulds. Correspondingly, detected pathogens were 25% Gram positive and 75% Gram negative.

#### Frequency of bacteria -detection in patients under immunosuppression

The SF test was positive for bacterial pathogens in 19/41 (46%) patients, whereas 6/41 (15%) cultures were positive (*p* = 0.004).

#### Frequency of fungal -detection in patients under immunosuppression

The SF test was positive for fungi in 6/41 (15%) patients, whereas 1/41 (2%) cultures were positive for fungi (*p* = 0.125).

#### Spectrum of detected pathogens in immunocompetent patients

**Fig. 2:**
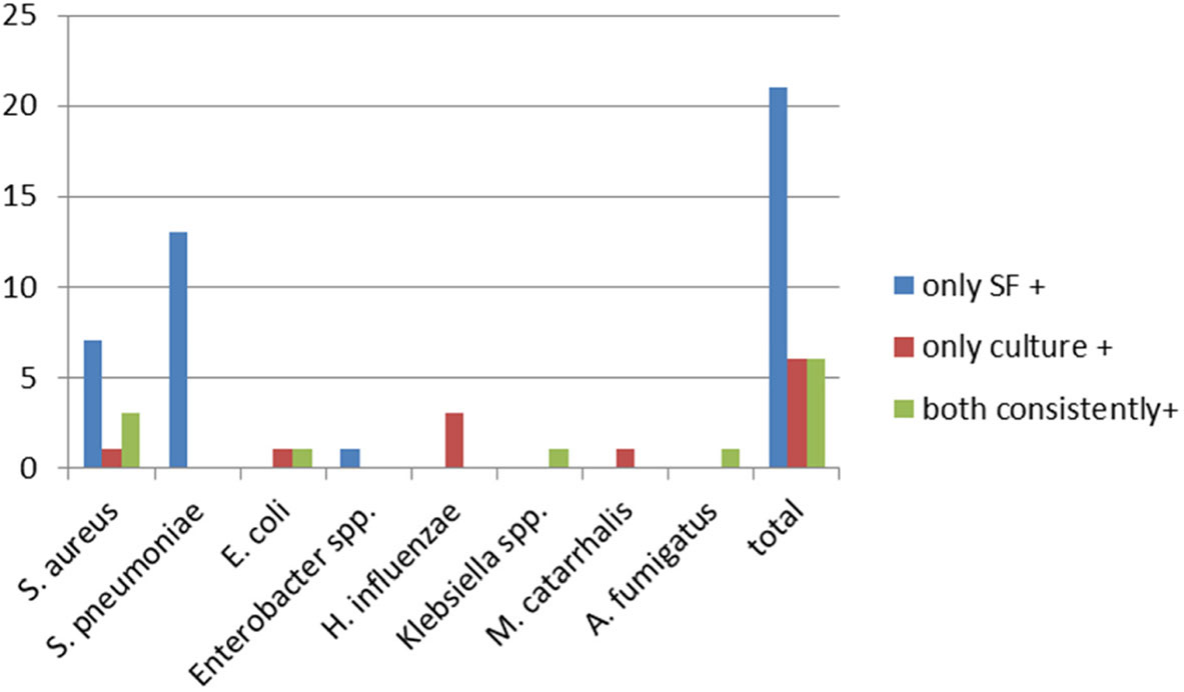
Spectrum of detected pathogens in immunocompetent patients.  only SF +, only culture +,  both consistently +

A total of 33 pathogens belonging to 8 different species were detected. Twenty-four of these pathogens were Gram positive, 8 were Gram negative and 1 was a mould (Fig. 2). Of the pathogens exclusively found by SF, 20 were Gram positive (95%) and 1 was Gram negative (5%). Of the pathogens exclusively positive by culture, 17% were Gram positive and 83% were Gram negative. Correspondingly, detected pathogens were 43% Gram positive, 43% Gram negative and 14% moulds.

#### Frequency of bacteria -detection in immunocompetent patients

The SF test was positive for bacteria in 25/29 (86%) patients, whereas 12/29 (41%) cultures were positive (*p* = 0.004).

### Pathogen detection in patients with antibacterial treatment vs. patients without antibacterial treatment

#### Frequency of pathogen detection in patients with antibacterial treatment vs. patients without antibacterial treatment

A total of 34 samples (49%) were taken from patients under antibacterial treatment. In this subgroup, pathogens were detected by the SF test in 47% (16/34) of samples and by culture in 6% (2/34) (*p* = 0.001). In untreated patients (*n* = 33), the SF test was positive in 76% (25/33) and the culture was positive in 45% (15/33) of the samples (*p* = 0.031).

#### Spectrum of detected pathogens in patients with antibacterial treatment

In all patients with antibacterial treatment, bacteria detected via SF were Gram positive (12 *S. pneumoniae* and 4 *S. aureus*).

## Discussion

This retrospective data analysis in a large paediatric cohort was carried out to investigate the detection rates of bacterial and fungal pathogens in BAL samples by the PCR-based molecular biological SF method compared to the standard culture method in children and young adults with pneumonia. We thought it was important to determine whether the application of the test was easily transferrable to material other than blood. We presumed that especially in patients with limited pathogen detection, e.g., after anti-infective pre-treatment, the addition of molecular biological methods with rapid results might have a substantial impact on the early detection of pathogenic bacteria and fungi.

We found that the sensitivity of the SF test for the frequency of pathogen detection is higher than that of culture, and the addition of the SF test enhances the pathogen detection rate nearly threefold. This enhancement is irrespective of the immunological status of the patient and applies to both bacteria and fungi. These findings correspond to what is known for the improvement of pathogen detection in bloodstream infections in children and neonates [9, 10, 24] and confirm initial results on the use of SF for pathogen detection from BAL fluid in adults [13] as well as the multiplex PCR -based diagnostics by Unyvero® (a multiplex PCR system for respiratory secretions detecting 20 bacterial pneumonia- causing microorganisms) from children and neonates [25].

It is well known that the cultivation of bacteria/fungi is rarely successful under antibiotic treatment [11, 26]. For bloodstream infections, preliminary data have proved SF to be more sensitive than culture. Pathogen detection has been enhanced 1.5-fold by SF. In particular, it is notable that in patients with antibiotic pre-treatment, the frequency of pathogen detectionincreased from 6.5–10% positive results by culture to 36% by SF [12, 27]. The first data of PCR-based pathogen detection from BAL fluid of pre-treated adults showed similar results: in pre-treated patients with a lower respiratory tract infection, pathogen detection was increased from 40 to 66% and from 23 to 64% using SF [13]. In particular, the detection of *S. pneumoniae* was increased from 2.9 to 31% by multiplex PCR [28]. Concordantly, the benefit of SF addition in our study cohort is even more striking for patients with antibacterial pre-treatment and for the detection of Gram positive bacteria. We increased the pathogen detection rate generally from 31 to 83% (2.7-fold) by SF and from 6 to 47% (7.8-fold) in pre-treated patients. In particular, the vast majority (86%) of Gram positive cocci in the entire cohort were only detected by the SF test. We also show at least a tendency for the detection of *A. fumigatus* to be improved by the addition of the SF test, which is of particular relevance, especially in immunosuppressed children. In immunosuppressed children, the therapeutic consequences are immediate and potentially life-saving. The advantage of PCR-based *Aspergillus* detection is already known for adults [16, 19, 29] and now, for the first time, has also been shown for paediatric patients.

As we present retrospective data, immediate consequences resulting from our findings cannot be stated. However, in pneumonia, the underlying pathogen is usually unknown, and even from the BAL fluid of these patients, pathogen detection is rare [6]. Our findings are helpful in elucidating the aetiology of pneumonia in children and providing information for differentiated antibiotic treatment. For all culture and SF results, contamination has to be taken into account; the percentage of contamination might be far higher than assumed here. The bronchoscope itself might be contaminated by oropharyngeal flora when passing the upper airways [30]. In particular, within the positive SF results contamination might be much more frequent than assumed: SF is a highly sensitive molecular method and even minor contamination could lead to a positive SF result. It is notable that the largest increases in species detection in SF vs. culture were amongst the Gram *positive upper airway colonisers S. pneumoniae* and *S. aureus*. As colonization of the oropharynx occurs early in childhood [31], both species of bacteria are prevalent in the upper airways of healthy children but can also be potential respiratory pathogens. Unfortunately, it is not feasible for us to determine what proportion of the increased detection in SF vs. culture is due to upper airway contamination. Such an analysis would require bronchoscopy and BAL sampling from healthy children, which would be difficult to justify. We only considered the PCR-based detection of microorganisms that usually show no pathogenicity to be false-positives. All other findings were assessed as positive related to our scientific question. At least all patients included in our study had suspected pneumonia. For everyday clinical practice, it is crucial for the evaluation of all results to include clinical information and should not be based on test results alone.

Knowing the pathogen load of detected microorganisms could theoretically help to estimate their pathogenic relevance. Unfortunately, multiplex PCR does not provide the option of quantification. This information might be misleading, especially in antibiotically pre-treated patients,because the pathogen load has already been reduced by therapy. Nevertheless, we must bear in mind that “overdiagnosis” by using a more sensitive method is an inherent problem, which again, makes the inclusion of careful clinical assessment indispensable. Our findings at least suggest that the SF test may be a useful additional diagnostic tool for pneumonia.

The SF test was originally established for blood testing but seems to also be applicable for BAL. However, important pathogens frequently causing pneumonia are not part of the SF spectrum. In the present study, the pathogen identification rate could have been even higher if the applied multiplex PCR had been designed for respiratory tract infections and included microorganisms frequently causing pneumonia, such as *H. influenzae*, *Mycoplasma spp., Chlamydophila spp.*, *P. jirovecii, and Legionella spp.* [32].

In 7 of 11 cases (64%) with a positive culture and negative SF result cultivated microorganisms were not part of the SF spectrum. Particularly in our cohort with more than half of the patients immunosuppressed, it was advantageous that *Aspergillus* was part of the assay panel spectrum. Ignoring the one patient with the *Paecilomyces* culture, which is not part of the SF spectrum, six of seven cases with *Aspergillus* detection would have been missed by cultivation alone. Applying the definitions of invasive fungal disease from the European Organization for Research and Treatment of Cancer/Invasive Fungal Infections Cooperative Group and the National Institute of Allergy and Infectious Diseases Mycoses Study Group (EORTC/MSG) Consensus Group [33], half of these cases (3/6) had probable, and another third (2/6) had possible aspergillosis. This finding underlines the relevance of the SF results from BAL for *Aspergillus* detection in immunosuppressed patients.

Knowing the limitations of the presented data, the findings are still significant and have clinical relevance in improving the sensitivity of aetiological diagnostics in children with pneumonia. The SF test provides quick results with potential therapeutic consequences. It would be worthwhile to confirm our data in a prospective evaluation of a larger cohort of patients also taking into account underlying immunosuppression, antibiotic pre-treatment, gain of time and concrete therapeutic consequences.

## Conclusion

In children with severe pneumonia, SF analysis of BAL provides increased pathogen identification rates and is therefore a useful tool as a supplement to BAL culture. The SF test cannot replace cultivation of the material because the pathogen spectrum is limited, and resistance testing cannot be carried out.

Future studies must prospectively and systematically compare both methods and scrutinize the benefit of detection and the influence on therapy and its relevance.

## Data Availability

The dataset used and analysed during the current study is available from the corresponding author on reasonable request.

## Abbreviations

ATS: American Thoracic Society
BAL: Bronchoalveolar lavage
CF: Cystic fibrosis
conS: Coagulase-negative-Staphylococci
f: Female
m: Male
MiQ: Quality standards of microbiological-infectiological diagnostics
No: Number
PCR: Polymerase chain reaction
SF: SeptiFast®
spp: Species
y: Years
